# Wayfinding the Live 5-2-1-0 Initiative—At the Intersection between Systems Thinking and Community-Based Childhood Obesity Prevention

**DOI:** 10.3390/ijerph13060614

**Published:** 2016-06-21

**Authors:** Shazhan Amed, Stephanie Shea, Susan Pinkney, Joan Wharf Higgins, Patti-Jean Naylor

**Affiliations:** 1 Department of Pediatrics, University of British Columbia, Vancouver, BC V6H 3V4, Canada; 2 Child & Family Research Institute, BC Children’s Hospital, Vancouver, BCV5Z 4H4, Canada; sshea@cfri.ca (S.S.); spinkney@cfri.ca (S.P.); 3 School of Exercise Science, Physical and Health Education, University of Victoria, Victoria, BC V8W 3P1, Canada; jwharfhi@uvic.ca (J.W.H.); pjnaylor@uvic.ca (P.-J.N.)

**Keywords:** childhood obesity, participatory research, health promotion, collective impact

## Abstract

Childhood obesity is complex and requires a ‘systems approach’ that collectively engages across multiple community settings. Sustainable Childhood Obesity Prevention through Community Engagement (SCOPE) has implemented Live 5-2-1-0—a multi-sector, multi-component childhood obesity prevention initiative informed by systems thinking and participatory research via an innovative knowledge translation (KT) model (RE-FRAME). This paper describes the protocol for implementing and evaluating RE-FRAME in two ‘existing’ (>2 years of implementation) and two ‘new’ Live 5-2-1-0 communities to understand how to facilitate and sustain systems/community-level change. In this mixed-methods study, RE-FRAME was implemented via online resources, webinars, a backbone organization (SCOPE) coordinating the initiative, and a linking system supporting KT. Qualitative and quantitative data were collected using surveys and stakeholder interviews, analyzed using thematic analysis and descriptive statistics, respectively. Existing communities described the consistency of Live 5-2-1-0 and extensive local partnerships/champions as catalysts for synergistic community-wide action; new communities felt that the simplicity of the message combined with the transfer of experiential learning would inform their own strategies and policies/programs to broadly disseminate Live 5-2-1-0. RE-FRAME effectively guided the refinement of the initiative and provided a framework upon which evaluation results described how to implement a community-based systems approach to childhood obesity prevention.

## 1. Introduction

Childhood obesity is a complex, far reaching problem that promises increasing rates of serious chronic disease like type 2 diabetes, heart disease, and even cancer. Complex social problems require ‘systems approaches’; multi-component, multi-setting adaptive solutions that are implemented in real-world settings [1,2]. Sustainable Childhood Obesity Prevention through Community Engagement (SCOPE) has developed and implemented the Live 5-2-1-0 initiative in partnership with British Columbian communities that are motivated to take community-wide action to promote and support healthy behaviours among children and their families. Live 5-2-1-0 is a multi-sector initiative that works with a wide range of community stakeholders to share (through social marketing) and support (through systems level change; programmatic, environmental, and policy level) the evidence based Live 5-2-1-0 message—at least five vegetables and fruits, <2 h of screen time, at least 1 h of physical activity, and zero sugary drinks, per day. This represents what Allender and colleagues describe as the “logical intersection” of systems thinking and community-based obesity prevention [3]; a recommended approach for engaging the complexity of both the problem of, and the solution to, childhood obesity [1].

SCOPE, the organization that coordinates the Live 5-2-1-0 initiative (referred to as ‘initiative’ from herein), is rooted in the principles of community based participatory research (CBPR) [4]. The initiative is informed by the socio-ecological framework [5] and requisite conditions of collective impact (a common agenda, mutually reinforcing activities, continuous communication, backbone support, and shared measurement) which is the ‘long term commitment of a group of important actors from different sectors to a common agenda for solving a specific social problem [6]’. Described in detail elsewhere [7], briefly the initiative’s common agenda, Live 5-2-1-0, is shared and supported across various sectors of a community achieving mutually reinforcing activities. SCOPE, the backbone organization, coordinates the initiative in multiple communities and provides the support needed for continuous communication between SCOPE and community stakeholders as well as between communities themselves. Lastly, SCOPE supports developmental and formative evaluation of the overall initiative through shared measurement via a novel shared data entry platform and a set of common process and outcome indicators.

SCOPE’s knowledge exchange (KE) model (Table 1) emerged in its first phase of development where SCOPE established a partnership with a local backbone organization (*i.e.*, local government, Division of Family Practice) in two pilot communities and through this partnership, a network of community-based, multi-sectoral partnerships was established via an intensive community engagement strategy that was rooted in the principles of community based participatory research [4], and relied heavily on ‘snowball recruitment’. This process of partnership development has been described in detail elsewhere [7,8]. Also, proof of concept initiatives that shared or supported Live 5-2-1-0 materialized as a result of these multi-sectoral partnerships and community specific action plans were developed that were tailored to the communities’ unique needs, strengths, and priorities for action. The outcome was a process for creating and maintaining these collaborative and respectful relationships and for supporting the progression from knowledge exchange to community action planning to sustainable action. Further, a ‘linking system’ [9,10] emerged that connected SCOPE to local knowledge users (*i.e.*, mayor and council members, health professionals, media outlets, public health officials, *etc.*) and supported continuous and bi-directional KE. Derived from the experiential learnings of this first phase of the initiative’s development and in response to the needs of community stakeholders, we adapted the RE-AIM model into our KT model which we called RE-FRAME (Table 1). RE-AIM [11] is a health promotion evaluation framework that has been designed for use in the assessment of the planning and implementation of initiatives aimed at addressing the knowledge to action gap [12]. RE-AIM has five key components (reach, effectiveness, adoption, implementation, and maintenance) and has informed our KE model with the objective of supporting the sustainability and deepening of the initiative in existing communities and facilitating effective and efficient KE. In this way, new communities interested in implementing the initiative could leverage existing knowledge while still having the opportunity to customize the initiative to their unique community context. For each of the components of RE-FRAME, indicators measuring Reach, Effectiveness, Adoption, Implementation, and/or Maintenance were identified.

In this paper we describe the protocol for implementing and evaluating the RE-FRAME model as an approach to understanding how to facilitate and sustain the beginnings of a systems-level/community-level change. Our mixed-methods study gathered data to assess the utility of the model in:

1. Supporting the development of meaningful partnerships and networks in new Live 5-2-1-0 communities;
2. Achieving sustained partnerships in existing Live 5-2-1-0 communities that are functioning with tangible results (*i.e.*, implementation of community action plans, development of new or enhancement of existing initiatives, or broadening multi-sector engagement); and
3. Transferring knowledge gained in SCOPE’s first phase between communities while still responding and adapting to complex, non-linear community dynamics by identifying: (i) the key knowledge that is necessary to share between communities (*i.e.*, sharing of common resources, collaborative program planning between Live 5-2-1-0 communities); and (ii) the model features that are best implemented with fidelity *vs.* those requiring modification to suit local conditions.

## 2. Materials and Methods

### 2.1. The KE Model

**Figure 1:**
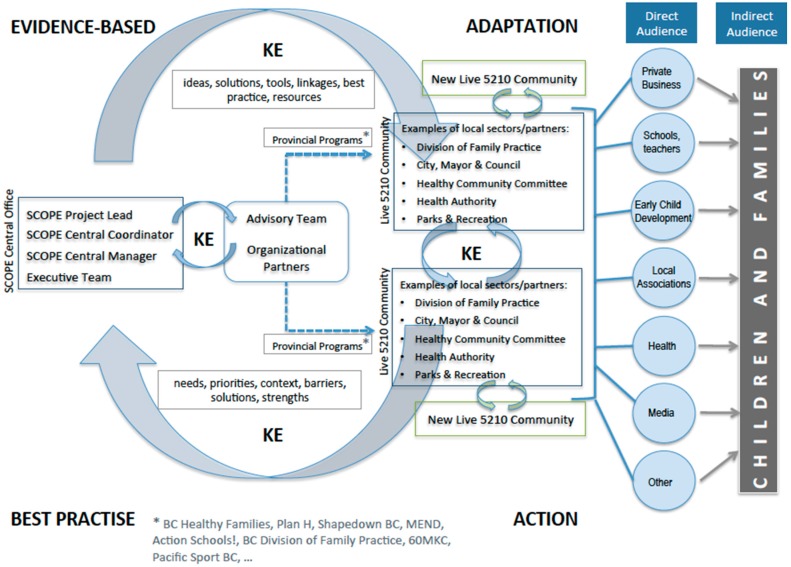
SCOPE’s linking system [7].

RE-FRAME is implemented through the following key components:

1. The Live 5-2-1-0 Online Resource Map [13] houses SCOPE’s resources (by sector) that can be downloaded for free and used across multiple community settings to share and support Live 5-2-1-0. These include videos, posters, fact sheets, newspaper/magazine advertisements, and a marketing guide to share the message; toolkits and checklists to build capacity among community stakeholders (*i.e.*, service providers, recreation facilitators, health professionals, and educators) to support the message; and ‘how to’ guides that outline key steps in action implementation to facilitate broader dissemination of innovative ideas. These resources represent a key outcome of SCOPE’s collaborative partnerships where they were conceptualized by community partners and their creation was supported by the SCOPE team who provided insights on best practice and strategies to align with regional and provincial programs as well as the financial support necessary to create high quality materials. (Resources; Facilitation, coaching and training)
2. Webinars are organized and held quarterly by the SCOPE team to provide the opportunity for new communities to interact with coordinators and stakeholders from existing Live 5-2-1-0 communities so that challenges and solutions to implementation, experiential learning, ongoing adaptation, and new ideas are shared. (Exchange of knowledge; Adaptation; Facilitation, coaching and training)
3. The SCOPE team (2 full time staff and the principal investigator) is the backbone organization that supports BC communities in sharing and supporting Live 5-2-1-0 and facilitates the alignment of community- and provincial-level activities. SCOPE provides coordination, facilitation and training, and resource development and/or adaptation while also supporting local and provincial stakeholder engagement, communications, and evaluation. The SCOPE team leads the implementation of the KE model through a comprehensive community engagement process that involves engaging with community champions and leaders who express interest in implementing the initiative, and coaching stakeholders as they proceed through the various stages of implementation. This is achieved via presentations to community groups representing multiple sectors (*i.e.*, healthy partnership table) or a single sector (*i.e.*, schools, health, community services), formal workshops, and practical continuous ad hoc communication via email and phone. SCOPE also mobilizes funding to support local coordination, action implementation, and evaluation. (Engagement; Facilitation, coaching, and training; Resources; Adaptation; Mobilization of champions; Exchange of knowledge)
4. A linking system (Figure 1) connects knowledge providers and knowledge users so that there is a ‘two-way’ exchange of knowledge. In our initiative, the central SCOPE team, advised by an executive of researchers and provincial- and community-level stakeholders, translate knowledge to community stakeholders (*i.e.*, ideas, solutions, tools, linkages, best practice, resources) but also receive knowledge on the needs, priorities, contexts, and strengths of the local community, as well as their ideas for innovative local action. The linking system supports a process of continuous tailoring to meet the needs of local settings, contexts, and priorities (Adaptation) and also supports opportunities for Exchange of knowledge and Facilitation, coaching and training. The linking system allows for the SCOPE team to advise community stakeholders on best-practice so that community-tailored messaging and/or initiatives are aligned with evidence-based principles. For example, in community C, an intervention using ‘nudge’ theory has recently been implemented in a local grocery store that was informed by the evidence-base [14] and is currently being evaluated in the local context.

### 2.2. Theoretical Framework

We used Graham *et al.*’s knowledge to action (KTA) framework [15] that is divided into knowledge creation and action, with fluid boundaries between each where they can occur simultaneously or sequentially and where they continually influence each other [15]. In our work, knowledge was initially derived empirically from previously published research demonstrating that a community-wide, multi-component approach to childhood obesity prevention results in improved health behaviours among children and decreased rates of unhealthy weights [16,17,18]. However, as our initiative crystallized, knowledge creation shifted to the experiential learning from ‘seeing and doing’, as well as the emergence of patterns that manifested through the self-organization of interacting sectors and stakeholders working collaboratively to develop a collective and collaborative action plan. The RE-FRAME model was a key knowledge tool that emerged in this first phase of SCOPE and has since been used to facilitate both the bi-directional transfer of knowledge between SCOPE and its community partners and between communities themselves.

**Figure 2:**
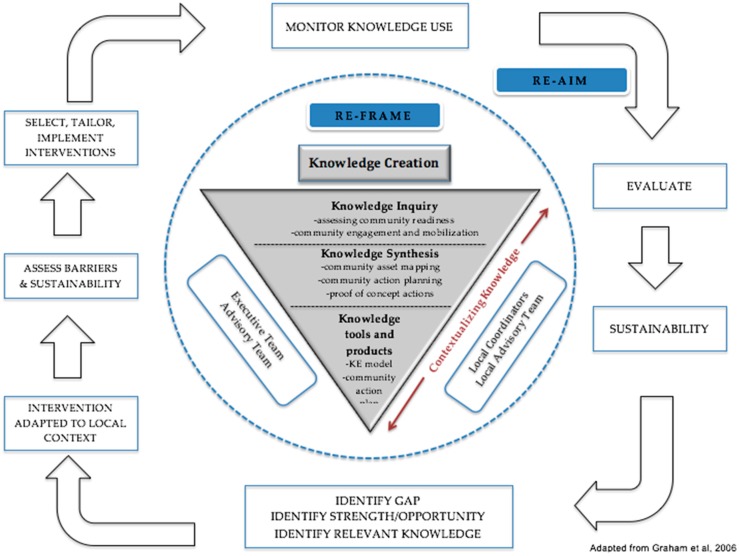
Knowledge to action cycle [15].

The action cycle (Figure 2) surrounding knowledge creation represents the application of the knowledge exchanged into community-level action that supports children in achieving healthier lifestyles. Key steps in progressing from knowledge to action include adaptation of the knowledge to the local context, identifying barriers and facilitators to knowledge use, building capacity among the stakeholders who interact with children (*i.e.*, educators, health professionals, private businesses, recreation facilitators, *etc.*), followed by implementation, evaluation and maintenance. The RE-FRAME model is designed to support these steps so that action implementation is planned and deliberate, rather than passive and unprepared, and is adaptive and responsive to changing community dynamics in order to optimize sustainability.

### 2.3. Participating Communities

Four BC communities were recruited to participate in this study—Communities A (C-A) and B (C-B) that have been implementing the Live 5-2-1-0 initiative since 2009 and 2012 respectively and are categorized as ‘existing’, and communities C (C-C) and D (C-D) that have more recently implemented the initiative in 2014 and are categorized as ‘new’. Communities A and B are large cities (population size 141,498 and 86,857 respectively) and C is comprised of several small municipalities (total population size 29,348) (Stats Canada); all three are adjacent to each other and within the same geographic region in BC. Community D is a small city (population size 6746) located in a more remote area of the province.

### 2.4. Data Collection and Analysis

Through the application of the KTA cycle (Figure 2) and using the RE-AIM framework [11], we developed a mixed-methods protocol to evaluate our KE model’s effectiveness in supporting the development and maintenance of multi-sectoral partnerships as well as the transfer and exchange of knowledge leading to community-wide action that shares and supports Live 5-2-1-0. The mixed-methods protocol was comprised of qualitative (*i.e.*, key informant interviews) and quantitative (web analytics, surveys, tracking tools, *etc.*) data. Our data collection tools are described below:

1. Partnership Tracking Tool (PTT): The PTT is a shared data collection and management tool that was established in SCOPE’s first phase and since then has been adapted multiple times in response to the feedback provided by community stakeholders on ease of use and usability of the data. Today, the PTT is a data collection platform that can be accessed online (with a username and password) by the SCOPE team and key stakeholders within Live 5-2-1-0 communities. The overall objective of the platform is to track new and existing community partners engaged in sharing and supporting Live 5-2-1-0. Data collected includes information on the partner organization (*i.e.*, sector represented), characteristics of the partnership (*i.e.*, when the partnership was established, stage of partnership), and outcomes of the partnership (actions implemented, Live 5-2-1-0 resources disseminated, *etc*.). Data on each partnership is updated so that the progress of partnerships over time is captured in terms of whether they evolve from action planning to action implementation, or lapse over time. The PTT data are analyzed bi-annually both in aggregate form and by community.
2. Community Capacity Building Tool (CCBT): The Public Health Agency of Canada’s (PHAC) CCBT is a valid, reliable tool that measures the community capacity building that occurs during the course of a project [19]. It measures change in community capacity over time with questions that cover nine domains (participation; leadership; community structures; role of external supports; asking why; obtaining resources; skills, knowledge, and learning; linking with others; sense of community). Responses are either yes/no or are on a four-point ordinal scale: (1) just started; (2) on the road; (3) nearly there; or (4) we’re there. Space is also available for free text to further describe each response. The CCBT was completed by each community at baseline with a plan to repeat the survey yearly for the duration of the project.
3. Surveys: Community stakeholders who participate in SCOPE’s KE initiatives (*i.e.*, webinars, workshops) are requested to complete a short survey asking about their satisfaction with the event, what aspects were most and/or least helpful, whether information received will influence action in their own community, and suggestions for improvement.
4. Semi-structured Interviews (*n* = 35) (Table S1): Local community coordinators who lead the Live 5-2-1-0 initiative within their communities (*n* = 4) and who are the main liaison to the SCOPE central team participated in semi-structured qualitative interviews at the start of the study with the plan to repeat these interviews yearly. A convenience sample of other community stakeholders who are involved in delivering the Live 5-2-1-0 initiative within their own organizations or sectors also participated in semi-structured interviews where questions assessed awareness of Live 5-2-1-0, the utility of Live 5-2-1-0 resources, facilitators and barriers to use, and resource adaptation. These stakeholders represented various sectors (education, health, community services, local government, recreation, media, early childhood, businesses) who were engaged in implementing Live 5-2-1-0 in their organization or local business (C-A: *n* = 6; C-B: *n* = 9; C-C: *n* = 6; C-D: *n* = 10). Qualitative interviews were conducted at baseline with a plan to repeat them annually for the duration of the project.
5. Web Analytics: The website [13] is monitored quarterly using web analytics where number of visitors to the resource page and resource downloads are tracked over time.

Quantitative data were analyzed using descriptive statistics (means, medians, proportions, standard deviations) and non-parametric tests of significance when appropriate. NVIVO Software was used to organize and analyze qualitative data using thematic analysis—grouping diverse sections of data into smaller analytic units [20]. For the purposes of this paper results from the partnership tracking tool, CCBT, local coordinator and stakeholder interviews, surveys, and web analytics were integrated in relation to their respective components within RE-FRAME model.

## 3. Results

### 3.1. Reach

Live 5-2-1-0 marketing resources and toolkits available on the Live 5-2-1-0 resource map [13] are a particular strength of the initiative, and are freely available to all stakeholders from all communities across BC and beyond. Over a 2-year period (2014–2016) the Live 5-2-1-0 resource map has had 373 unique users from 68 unique communities across BC and a total of 2371 Live 5-2-1-0 resources have been downloaded. A subset of individuals (*n* = 58) who downloaded resources from the Live 5-2-1-0 Resource Map were contacted by email and asked how they used the resources and what was happening in their broader community related to Live 5-2-1-0. Eighteen (31%) downloaders responded representing various sectors including schools/afterschool (*n* = 4), health (*n* = 7), early childhood programs (*n* = 3), recreation (*n* = 4) and other (*n* = 1). Respondents were using the resources to share the message; for example an independent ventriloquist integrated Live 5-2-1-0 into a show promoting healthy behaviours and a family doctor hung the 52 tips Live 5-2-1-0 poster in his clinic’s play area with a new tip displayed every week. In a local health unit “nurses use these resources during children’s immunization clinics (where) they will go over the information with parents…” The live 5-2-1-0 goal tracker was being used in an afterschool program and early childhood and recreations programs were sharing the message in their newsletters. Many downloaders were introducing the resources to community partners in order to get buy-in to use Live 5-2-1-0 more broadly and to implement action (*i.e.*, Live 5-2-1-0 Playboxes) to support the message.

Data extracted from the PTT illustrates number and progression of partnerships and also provides estimates of the number of Live 5-2-1-0 resources distributed and actions implemented related to sharing or supporting the message. Due to recent staff changeover in community D, data has not been entered into the PTT and therefore was not available. From 2013 to 2015, communities A and B distributed 24,431 and 11,468 Live 5-2-1-0 resources respectively and in 2015, community C distributed 1029 resources. Across all three communities, 67 different community organizations or groups distributed Live 5-2-1-0 resources such as health care providers, not-for-profit organizations, schools, chamber of commerce, private businesses, media, and the local health authority. Resources may have been distributed to the same people or partners over this time period.

**Figure 3:**
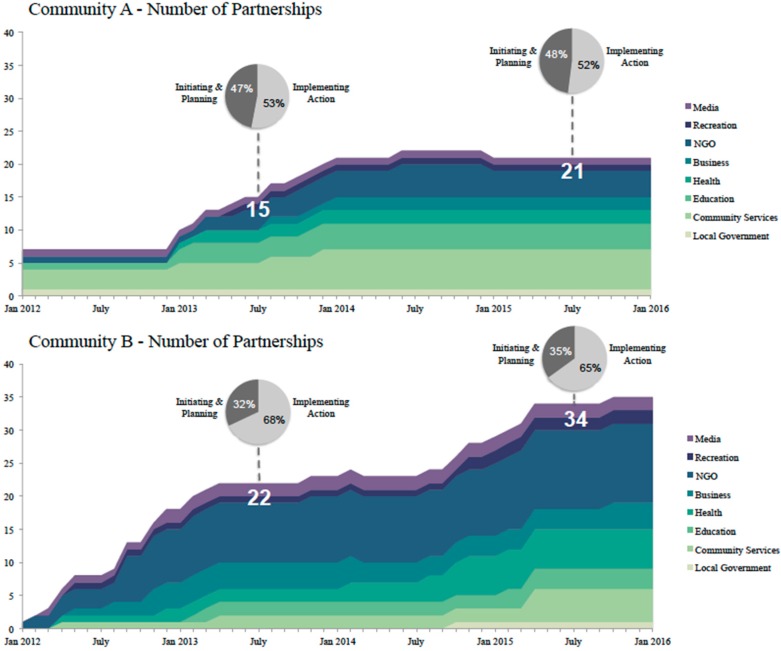
Progression of partnerships in existing Live 5-2-1-0 communities A and B.

The number and progression of partnerships is illustrated in Figure 3 for existing Live 5-2-1-0 communities and Table 2 outlines the number of actions related to Live 5-2-1-0 implemented by community. In community A, 7/15 partners in July 2013 were in the planning stage; by July 2015, two of those seven partners had moved into implementing action, while five remained in the planning stage. Of the six additional local partners that had joined the initiative by July 2015, one of those had already moved into the action stage, while five of those new partners were still in the planning stage. In community B, 7/22 partners in July 2013 were in the planning stage; two of those seven partners had moved into the action stage by July 2015, while four remained at the planning stage and one partnership had been suspended. Thirteen additional partners joined the initiative between July 2013 and July 2015, of which five moved from planning to action, while eight remained in the planning stage by 2015.

### 3.2. Resources

Overall, new and existing Live 5-2-1-0 communities had adequate capacity and resources to implement Live 5-2-1-0, although sufficient funding was an ongoing challenge. Existing Live 5-2-1-0 communities were further down the road in the CCBT domains of participation, leadership, community structures, and asking why (Table 3) as compared to new Live 5-2-1-0 communities. However, both existing and new communities similarly reported having sufficient internal (*i.e.*, community level leadership, volunteers, in-kind contributions from local organizations and businesses) and external (Live 5-2-1-0 online resources, SCOPE central office support, health authority support, and funding) resources, indicating that new communities were well supported at the start of implementing Live 5-2-1-0 and existing communities had sustained support, both from within and outside of their community.

In existing communities, almost all of the available online Live 5-2-1-0 resources were being used however newer communities were mostly using marketing resources to share Live 5-2-1-0. Overall, the resources were described by stakeholders as being easy to use and integrate into existing programming. The simplicity of the message and the ‘eye catching’ colourful graphics were identified as strengths, as well as the simple content that made the resources usable with a broad range of audiences. As one of the interviewees in Community C noted, “It’s easy to read, it doesn’t have a lot of literature on it, but it’s specific. So it’s got enough detail on it but not too much. The literacy level on it is about grade 9 which is perfect…, it’s attractive to the eye and the messaging is clear.” (C-D7) 

Live 5-2-1-0 resources were also described as being easy to adapt to local contexts. Existing communities were identified as ‘resource creators’ and new communities as ‘resource adaptors.’ The content of the resources remained largely the same when implemented across different communities. For example, as a Community B stakeholder explained: “It’s not a lot of content change with the exception of putting the local lens on it. I would say it’s more so the branding logo changes.” (C-B)

### 3.3. Engagement and Mobilization of Champions

A first level content analysis of the local coordinator interviews focused on baseline similarities and differences across existing *vs.* new communities related to factors that drove the process of engaging stakeholders and mobilizing champions. Communities identified their own unique strategies for engaging community stakeholders. Existing communities described the importance of partnerships and a collective approach in reaching the broader community whereas new communities focused on leveraging community champions and holding networking events to drive the engagement process.

- “Our experience here in (community A), we really need to have a common cause, a common project, something that we’re working on with our partners to keep them engaged.” (C-A)
- “We’re keeping an actively engaged community that already has a really strong table for child and youth issues and an understanding of group organizations.” (C-B)
- “…just finding somebody who truly believes in the message so that you have a champion…, if you have somebody that is a champion then you will get really far but if you don’t have that champion in place, it sort of stalls the whole progress.” (C-C)

Local champions were described as being critical for creating local connections: “It’s having the connections within your own organization and the passion about the specific topic.” (C-C). Little effort was needed to engage champions however, aligning efforts with champions and providing them with capacity to implement the initiative is important: “They are actively looking for opportunities for the 5-2-1-0 message to be further integrated and shared in our community…, they’re the ‘ideas’ people.” (C-A) 

For both new and existing communities, stability and sustainability of partnerships was identified by local coordinators as critically important: “I think the number one thing (with partnership sustainability) is consistency of the representatives…, the sustainability really hinges on having that consistent representation.” (C-A). Developing, fostering, and maintaining partnerships requires frequent face to face conversations and a significant investment of time: “There’s been a lot of hours invested in just creating a relationship with these organizations. And that relationship-building part is absolutely the most crucial factor.” (C-A). Newer communities identified financial resources as a barrier to achieving community engagement however, in existing communities where the initiative is more institutionalized, time, more than money, was a critical factor in maintaining the momentum of partnerships.

Ongoing efforts to engage stakeholders and sustain partnerships was key to achieving community-wide awareness of Live 5-2-1-0 where in existing communities A and B, awareness was high among stakeholders who described it being ‘all over’ their community: “Everywhere, I have seen it lots. I see it in the schools, I see it at different events in the community, and I have seen it in the newspaper, lots of different places.” (C-B). In new communities, stakeholder awareness of Live 5-2-1-0 was predominantly through a local ‘launch’ event (*i.e.*, Health Fair in C-C) or via the community’s healthy partnership table.

### 3.4. Facilitation, Coaching and Training, and Exchange of Knowledge

Facilitation and coaching provided by SCOPE central office and local coordinators in Live 5-2-1-0 communities to community stakeholders combined with high-quality Live 5-2-1-0 resources and formal opportunities for knowledge exchange (*i.e.*, webinars) have provided the necessary capacity for community stakeholders to implement Live 5-2-1-0 in their local communities. Knowledge exchange between SCOPE central office and local stakeholders had been predominantly ad hoc coaching via meetings, presentations, email, and telephone. However, more recently, three formal webinars were coordinated by the SCOPE central office with a total of 37 participants representing 12 communities across BC. The focus of these webinars was to provide opportunities for knowledge exchange between communities. The webinars covered key topics such as answers to frequently asked questions from other communities in BC interested in implementing Live 5-2-1-0; how to conduct community assessments and evaluation; and how initiatives supporting Live 5-2-1-0 implemented in existing communities could be ‘cross implemented’ in new communities.

Fifty percent (19/37) of participants completed the survey and all reported being satisfied or very satisfied with the overall quality of the webinars; 90% found the webinars helpful in enhancing their capacity to share and support Live 5-2-1-0 in their local communities. Two-thirds (63%) of respondents reported that the information they received from the webinar would influence how they implement Live 5-2-1-0 in their community and provided examples such as: “it gave practical ideas for communities early on in the process on how to effectively engage (stakeholders)”; and “helped us ask whether we have the right people in the room and ensuring that the involvement is broad enough.” Participants appreciated the “conversational” style of the webinars, highly valued the perspectives of and connections to local coordinators from existing Live 5-2-1-0 communities, and appreciated hearing the “concrete examples of actions” that had been implemented.

Stakeholder interviews confirmed that the facilitation and coaching, Live 5-2-1-0 resources, and opportunities for knowledge exchange resulted in broad community awareness and action to promote healthy childhood behaviours. All stakeholders from communities A and B were sharing Live 5-2-1-0 and only one reported the need for additional support. Almost all stakeholders in new and existing communities felt confident in sharing and supporting Live 5-2-1-0 because of the simplicity of the message, the easily accessible resources, and the extensive support provided by SCOPE central office and its local community partner organizations. The community-wide approach of the Live 5-2-1-0 initiative was also identified as a strength.

- “I love that it is a simple message and that it is very concise and easy to do…., and I think because it’s a community initiative, the piece for me is that it makes sense to do it because other people are doing it as well.” (C-D)
- “…yes we feel very confident in the messaging. It’s very simple and straightforward and the toolkit has really helped give staff the resources to deliver that message…” (C-A)
- “I do feel confident in implementing changes and I believe my fellow staff members feel confident. I think part of the reason we feel so confident is that we’ve gotten such excellent support from staff at your agency (SCOPE) and the materials you have provided us have given us excellent tools to work with.” (C-B)
- “I do feel confident sharing the message but I’m also very happy that the (SCOPE community partner organization) staff have been able to provide us with the support with staff that can actually come in and share the message. Whenever I’ve had questions about ways that I can creatively implement the message in my day-to-day programming, the staff has been available…” (C-B)

The Live 5-2-1-0 initiative also supported local stakeholders in transferring knowledge on healthy behaviours to the children and families with whom they interact and has provided them with the tools they need to institutionalize Live 5-2-1-0 into their daily practice. For example, in existing communities, Live 5-2-1-0 was shared by integrating the message in recreation programming (C-A), through community-based programs, and via events, websites, and through the use of marketing materials (*i.e.*, posters, newsletters, brochures). In community B, the local radio station was sharing the message on-air twice daily by providing in-kind advertising time. Stakeholders also reported supporting Live 5-2-1-0 by holding challenges centered around the goals of Live 5-2-1-0, and serving healthy snacks and integrating regular physical activity in their programs: “…we’ve had messaging around getting kids more physically active. So we would have weekly park visits in the summer and when we would do those visits we would actually say, “And this is part of 5-2-1-0.” So in our regular activities, when we serve snacks at Family Place for example, we will mention, “Kids should have and all of us should have this many fruits and vegetables in a day.” We incorporate the message in our regular programming when we’re explaining why we are doing something, it’s part of 5-2-1-0.” (C-B). Additional support for existing Live 5-2-1-0 communities is needed in the form of a more formal training curriculum for ‘on the ground’ community stakeholders as the capacity of SCOPE’s local partner organizations to support the rapidly growing interest in using Live 5-2-1-0 across their community is insufficient. Also, upstream social determinants of health were identified as a consistent challenge in families achieving the goals of 5-2-1-0.

Newer communities C and D were mostly sharing the message using marketing materials; limited to one or two community sectors (*i.e.*, recreation center, library, *etc.*) and community events. Live 5-2-1-0 was also being shared at partnerships tables comprised of various key stakeholders who are responsible for implementing child and youth programming. These newer communities expressed a need for funding support, materials and “learning from the experience of other communities that have done this and just general guidance on the best ways to make this happen and succeed in our community.” (C-D)

## 4. Discussion

The Live 5-2-1-0 initiative is a community-based intervention that uses a multi-strategy, multi-level approach to childhood obesity prevention in order to address the complexity of causes from the individual through to environmental and policy settings. Our early experience in implementing this initiative revealed key factors necessary for effective community engagement and partnership development that leads to coordinated local, sustainable action across multiple community settings. We summarized these factors into the RE-FRAME model that has since guided our implementation and evaluation efforts in two existing and two new Live 5-2-1-0 communities. Analysis of our baseline results has captured recurring patterns across four Live 5-2-1-0 communities and has also distinguished between communities based on length of tenure with Live 5-2-1-0.

In the more experienced communities, successful implementation of Live 5-2-1-0 was characterized by (1) consistent—that is reliable, regular and coherent—use of Live 5-2-1-0 communication and campaign resources; (2) a synergy among compatible existing initiatives and partners to promote healthy living across multiple settings using a common agenda; and (3) over time, the pervasiveness of the Live 5-2-1-0 message throughout the community. Existing communities A and B, effectively supported by SCOPE central office via coaching and resources, also relied heavily on their extensive local partnerships and champions, to disseminate and utilize Live 5-2-1-0 to create healthier environments for children and families. As a result, there was minimal adaptation of Live 5-2-1-0 materials beyond integrating different partner logos, although interviewees offered innovative recommendations to modify the initiative for their local context (e.g., to reduce handing out yet another ‘pamphlet’ and rather creating a Live 5-2-1-0 Storybook).

In the newer communities, the experience of implementing Live 5-2-1-0 was—not surprisingly —still fledging and finding its way. Despite an overall lower awareness and less sharing of the message, participants cited its credible and simple message as important attributes of their ability to feel confident in adopting and integrating it into local efforts. As well, participants recognized the compatibility of the message with other local campaigns that made it relatively straightforward to engage local healthy living leaders and settings as vehicles to begin its institutionalization. Communities C and D were looking to learn from the experiences of communities A and B to inform their own strategies and policies/programs, as well as for continued funding to further establish Live 5-2-1-0 materials and activities within local ethos.

The effect of community-based childhood obesity prevention initiatives on outcomes such as body mass index, rates of overweight and obesity, and child health behaviours has been previously described [16,18] however, details on how to implement, and importantly how to evaluate the implementation process of these community-wide interventions is not as readily available. Our goal, through this work, is to fill this knowledge gap on how a community can be engaged across multiple settings to participate in a collective and coordinated approach to achieving healthy childhood weights. RE-FRAME has served two key functions: first, it has guided the refinement and further development of the key elements of the initiative (*i.e.*, Live 5-2-1-0 online resources, webinars, linking system, SCOPE central office support, *etc.*) and second, it has provided a framework upon which our mixed-methods evaluation protocol was designed and analyzed. RE-FRAME afforded us the scaffold to describe the layered implementation processes of the Live 5-2-1-0 intervention with the intent to guide replication and/or adoption elsewhere.

We purposively tailored RE-FRAME from a widely used and established population health evaluation framework (RE-AIM) [11,21], integrating key experiential learnings from our early implementation experience rooted in the principles of community participation. As a result, our findings deliberately capture the perspectives of community stakeholders who are integral partners in the implementation of the Live 5-2-1-0 initiative, providing information that is critical to refining and improving the initiative. Further, RE-FRAME encompasses the concept of ‘adaptation’ which is critical to consider when scaling up an initiative such as Live 5-2-1-0 to inform resource needs and implementation processes in new communities, fueling the knowledge to action cycle (Figure 2). A key strength of our initiative is the ability to tailor the message to the unique needs of communities. Our use of RE-FRAME demonstrates how data can be used to improve an initiative, rather than prove the success of the initiative’s efficacy/effectiveness.

Our evaluation results can also be used to maintain engagement of and momentum among local stakeholders. Community-specific data that quantify and characterize partnerships complemented by qualitative ‘stories’ serve as a catalyst to build community capacity, increase momentum and deepen the work of local stakeholders by demonstrating the impact of their time and effort. The data also support continuous learning, quality improvement, and adaptive strategic planning. For example, in existing communities, the number of partnerships increased over time however, in SCOPE’s longest partnered community (C-A), new development of partnerships has plateaued (Figure 3), yet action implementation has been sustained (Table 2). As we continue our evaluation efforts, we will seek to understand whether this slowing of partnership development is the result of saturation; related to insufficient capacity to support new partnerships because of extensive adoption of Live 5-2-1-0 without added local capacity; or a loss of interest among community stakeholders.

Our key learnings emphasize the dynamic process of implementing and evaluating a community-based intervention that requires methodology that is emergent and adaptive in order to respond to changing community dynamics and stakeholder needs and feedback. We will repeat our data collection 1-year after collecting baseline data, however, our methodology has been adapted to minimize data collection burden and increase the likelihood of collecting evidence of change. For example, rather than repeating qualitative interviews with local coordinators, we will use the Most Significant Change (MSC) [22] method to effectively draw out observed and experienced ‘stories of change’ [23]. Interviews with broader community stakeholders will only be repeated in new communities where we will further characterize the experiences of these communities in implementing the Live 5-2-1-0 initiative with a focus on the utility of newly developed Live 5-2-1-0 resources, knowledge transfer from existing communities, and the process of adaptation. The PTT will continue to be adapted to enhance ease of data entry for local coordinators, as well as optimize data quality and usability. Opportunities to efficiently scale-up the PTT to expand our shared measurement across more communities implementing Live 5-2-1-0 will be explored (*i.e.*, converting the PTT to an App).

The overall initiative will also be augmented in response to our baseline results. Resource development will include a ‘Live 5-2-1-0 roadmap’ that will support local champions in navigating the process of implementation and aligning their existing efforts with the initiative, pre-made presentation slides that can be used by local champions to engage a broad range of community stakeholders across a wide range of sectors, and a formal Live 5-2-1-0 training curriculum for local stakeholders who directly interact with children and families. Social determinants of health continue to be a major barrier and SCOPE, in partnership with local stakeholders, must continue to develop, implement, and evaluate strategies that effectively address these broader determinants. Lastly, a scale up model that leverages regional infrastructure of existing provincial organizations/programs invested in promoting healthy childhood behaviours and weights will be explored to efficiently augment capacity in current Live 5-2-1-0 communities and extend our reach to new communities.

Although beyond the scope of our resources and expertise, we recognize that other models may also be of use to others interested in evaluating similar efforts offering more sophisticated measures and analytics of complex community relationships and structures. Already well established in public and population health research, social network analysis [24] maps the relationships and/or ties among organizations to reveal social structures of health phenomena, and system dynamic models [25] which apply the processes of accumulation and feedback to causal structures and policy inputs. Both approaches use computer simulated algorithms to depict the complexity and dynamics underlying system influences on health. As well, the more recent pragmatic Stepped Wedge Cluster RCT design [26] for evaluating ‘service delivery type interventions’ is a viable approach when there are logistical, ethical or political issues that preclude the use of traditional RCT designs. Stepped Wedge Cluster RCTs allow for the sequential allocation of participants to the intervention so that each cluster serves as both a control and an intervention. We must also acknowledge the lack of measures for primary health outcomes—body weight and related health behaviours. Finally, while our intent is to prevent childhood obesity informed by an ecological and systems perspective, we know that our experience described here reflect modest and early efforts.

## 5. Conclusions

SCOPE set out to address one of the most prominent “wicked problems” that scourge population health research and practice. Such problems demand innovative, iterative, comprehensive solutions that can be modified in light of experience and on-the-ground feedback. Usually, the solution involves changing behaviour; always it demands engaging citizens and stakeholders in its design and implementation [27]. Further, creating ecological, multi-level interventions is recognized as the most powerful approach to achieve significant change, the cost-effectiveness of which is significantly contingent on uptake [28,29]. A consistent rallying call within population health reminds us that “If we want more evidenced-based practice, then we need more practice-based evidence” [30]. In fact, few rigorously evaluated ‘gold standard’ interventions in public health achieve wide spread adoption, exacerbating the difficulty of adopting “best practices” that simply do not meet community needs, oversimplify community realities, or do not meet the requirement of ‘scalable efficiency [31,32]’. In our estimate, RE-FRAME works toward heeding this call as a multi-layered tool that repositioned evaluation from a focus on outcomes to understanding the reach of complex community initiatives and the processes that support them. In doing so, it offered us a KT version of wayfinding that led us to the intersection of systems thinking and community-based childhood obesity prevention.

## Figures and Tables

**Table 1:** The RE-FRAME knowledge exchange model.

Model Component	Description
Reach	The extent of the key players, partnerships, and collaborations that are actively participating in the development and implementation of the project
Engagement	Developing, sustaining and fostering relationships that facilitate knowledge exchange and sharing
Facilitation, coaching, training	Technical support and sharing of expertise through active participation of knowledge-users and on-site coaching
Resources	Development of new or contextualization of existing resources to enhance self-efficacy and skills around administering childhood obesity prevention initiatives
Adaptation	Continuous tailoring and adapting of activities to local settings, contexts, needs and priorities
Mobilization of champions	Identifying and mobilizing key champions and early adopters who represent various community sectors
Exchange of knowledge	Multiple levels of continuous, bi-directional exchange of knowledge, learning, and expertise

**Table 2:** Action implemented related to sharing or supporting Live 5-2-1-0.

	Existing Communities								New Community
	C-A				C-B				C-C
	2012	2013	2014	2015	2012	2013	2014	2015	2015
Community event	--	12	3	7	14	19	11	22	28
Community presentation	--	4	3	6	2	6	9	18	15
Policy/practice change	--	--	2	--	--	--	2	4	1
Stakeholder engagement	2	14	8	3	12	16	11	11	6
Resource development/adaptation	--	--	--	--	--	1	3	4	--
Environmental change	--	--	1	2	--	1	--	8	--
Funding	--	--	--	--	6	14	3	6	2
Training Workshop	--	3	4	2	--	--	6	2	3
Other	--	2	--	--	3	9	--	1	4
Total	2	35	21	21	37	66	45	76	59

**Table 3:** Baseline results of community capacity building tool (CCBT).

	Mean Score *			
	Existing		New	
CCBT Domain (Description) Community:	A	B	C	D
Participation (active involvement of community stakeholders)	3.00	3.75	2.25	2.00
Leadership (engagement of and support from formal and informal local leaders)	3.33	2.00	1.33	2.33
Community structures (engagement of community groups and committees)	2.33	3.00	1.67	1.67
Role of external supports (support from local government, foundations, or regional health authorities)	3.33	3.33	2.75	3.25
Asking why (a process for uncovering root causes for community health issues and potential solutions)	2.67	4.00	2.33	1.33
Obtaining resources (finding time, money, leadership, volunteers, and information from both inside and outside the community)	3.50	4.00	4.00	4.00
Skills, knowledge, learning (qualities in the project team and broader community stakeholders)	2.50	3.50	2.50	3.00
Links with others (links with individuals and organizations through partnerships, networks, and coalitions)	3.25	4.00	2.50	2.50
Sense of community (People coming together to work on shared community problems through collaboration)	3.00	4.00	2.00	3.00

* 1—Just started; 2—On the road; 3—Nearly there; 4—We’re there.

## Supplementary Materials

The following are available online at www.mdpi.com/1660-4601/13/6/614/s1, Table S1: Local coordinator and stakeholder interview questions.

## Abbreviation

The following abbreviations are used in this manuscript:

SCOPE	Sustainable Childhood Obesity Prevention through Community Engagement
