# Supplementary material for: Wayfinding the Live 5-2-1-0 Initiative—At the Intersection between Systems Thinking and Community-Based Childhood Obesity Prevention
Source: Int J Environ Res Public Health. 2016 Jun 21;13(6):614. doi: 10.3390/ijerph13060614 (PMC4924071; doi:10.3390/ijerph13060614)
Supplement: Supplementary file 1 [file ijerph-13-00614-s001.pdf]

Supplementary Materials: Wayfinding the Live 5-2-1-0 Initiative—At the Intersection between Systems Thinking and Community-Based Childhood Obesity Prevention

Shazhan Amed, Stephanie Shea, Susan Pinkney, Joan Wharf Higgins and Patti-Jean Naylor

Table S1. Local coordinator and stakeholder interview questions.

Local Coordinator Interview Questions
1. Which partners/organizations do you have the most effective partnerships with? Why is this? What is the frequency of contact with these partners? (daily, weekly, monthly, quarterly, annually).
2. What factors do you think have contributed to making partnerships in your particular community strong/weak?
3. What have been the most valuable achievements that have come about as a result of your partnerships?
4. Thinking about your total existing partnerships, what factors impact the sustainability of those partnerships? *(where sustainable means that you anticipate the partnership will continue for 1 year or longer)?*
5. Why do you think those particular partnerships have been sustained? (Probe: Are there any common qualities?)
6. Why do you think other partnerships have not been sustained?
7. From the sectors you've engaged through your partnerships:
8. Why do you think some sectors have partnered and other sectors haven't? (Probe: What facilitators are there for partnered sectors? What barriers exist for the sectors that have not partnered as much?)
9. We would like explore and track how one Live 5-2-1-0 partnership opens the doors to another Live 5-2-1-0 partnership(s) (*i.e., that snowball effect of partnerships coming from other partnerships*). Would you be able to take us through "the story" of how your partnerships are connected (if they are)?
10. Have your partnerships advanced your progress in promoting healthy childhood weights in your community? And if so, how? What would your progress have been without these partnerships?
11. On a scale of 1–5 where 1 is no impact and 5 is significant impact, Did your partnership with SCOPE impact partnership development in your community? Please explain your rating.
12. Are there any other important details about partners and partnerships that you think are not captured by the information recorded in the PTT?
13. What are the main facilitators to developing partnerships and relationships and maintenance of those partnerships?
14. What are the main barriers to developing partnerships and maintenance of those partnerships?
15. What are the most important things you've learned about developing and maintaining partnerships/relationships in your community?
16. What Live 5-2-1-0 resources have you created and implemented in your community? What resources have you adapted and implemented in your community that were created elsewhere?
17. How have you adapted the Live 5-2-1-0 resources to meet the unique needs of your community?
18. Which Live 5-2-1-0 resources have been particularly useful in your community (What has not been useful?)
19. Have there been any facilitators and barriers to disseminating and using the resources?
20. In your opinion, how should the new or existing Live 5-2-1-0 resources be changed or

expanded to further enhance your partners' self-efficacy and skills in promoting healthy weights in kids in your community?

21. Why did you identify these individuals as champions? (Probe: What 'champion-like' qualities do they possess? What is their role in the community?) How do they promote and support the Live 5-2-1-0 message and initiatives?

Stakeholder Interview Questions

1. Where have you seen or heard about the Live 5-2-1-0 health promotion message?
2. Have you shared the Live 5-2-1-0 message through your services and/or programs?
3. We really appreciate partners sharing and communicating the Live 5-2-1-0 message. Could you tell me about how you have shared the Live 5-2-1-0 message and/or materials?
4. Would you/your team want to share this message if there was support? Please explain what this support would look like to you.
5. Do you feel confident and/or able to share and communicate the Live 5-2-1-0 message to the children and families you work with? Why/Why not?
6. Have there been any actions taken or changes made (such as changes to programs, policies, or the environment) within your organization to support or make it easier for children and families in Living 5-2-1-0?
7. Do you feel confident and/or able to implement changes to support Live 5-2-1-0 within your organization? Why/Why not?
8. Could you tell me what Live 5-2-1-0 materials/resources you have used in your services/programs? And how you have used them?
9. Are the Live 5-2-1-0 materials easy to integrate with the children and families you work with?
10. Did you adapt or change the Live 5-2-1-0 resources/materials to fit your community context or your specific programs?
11. Are there others you know in the community that we should have this conversation with?

© 2016 by the authors. Submitted for possible open access publication under the terms and conditions of the Creative Commons Attribution (CC-BY) license (http://creativecommons.org/licenses/by/4.0/).
